# Sutureless aortic valve for reoperative aortic valve replacement as an alternative to composite graft replacement

**DOI:** 10.1016/j.xjtc.2024.01.007

**Published:** 2024-01-18

**Authors:** Taisuke Nakayama, Yoshitsugu Nakamura, Yuto Yasumoto, Borut Gersak

**Affiliations:** a Department of Cardiovascular Surgery, Chiba-Nishi General Hospital, Chiba, Japan; b University of Ljubljana School of Medicine, Ljubljana, Slovenia

**Figure:**
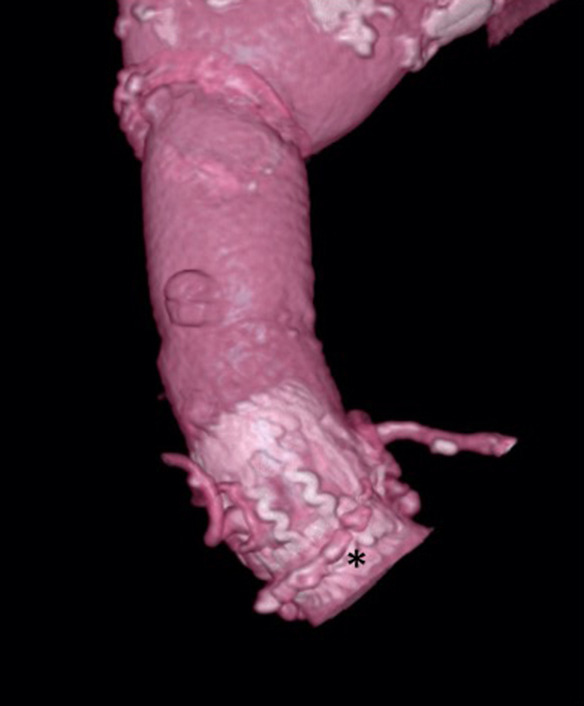
Postoperative CT image of SAV and aortic root. ∗Previous surgery pledgets.

**Central Message**

A successful redo AVR procedure with a SAV was performed in a patient who had previously undergone Bentall surgery. The cuff and pledget of the previous prosthetic valve were left in place.

## Case Presentation

**Figure 1:**
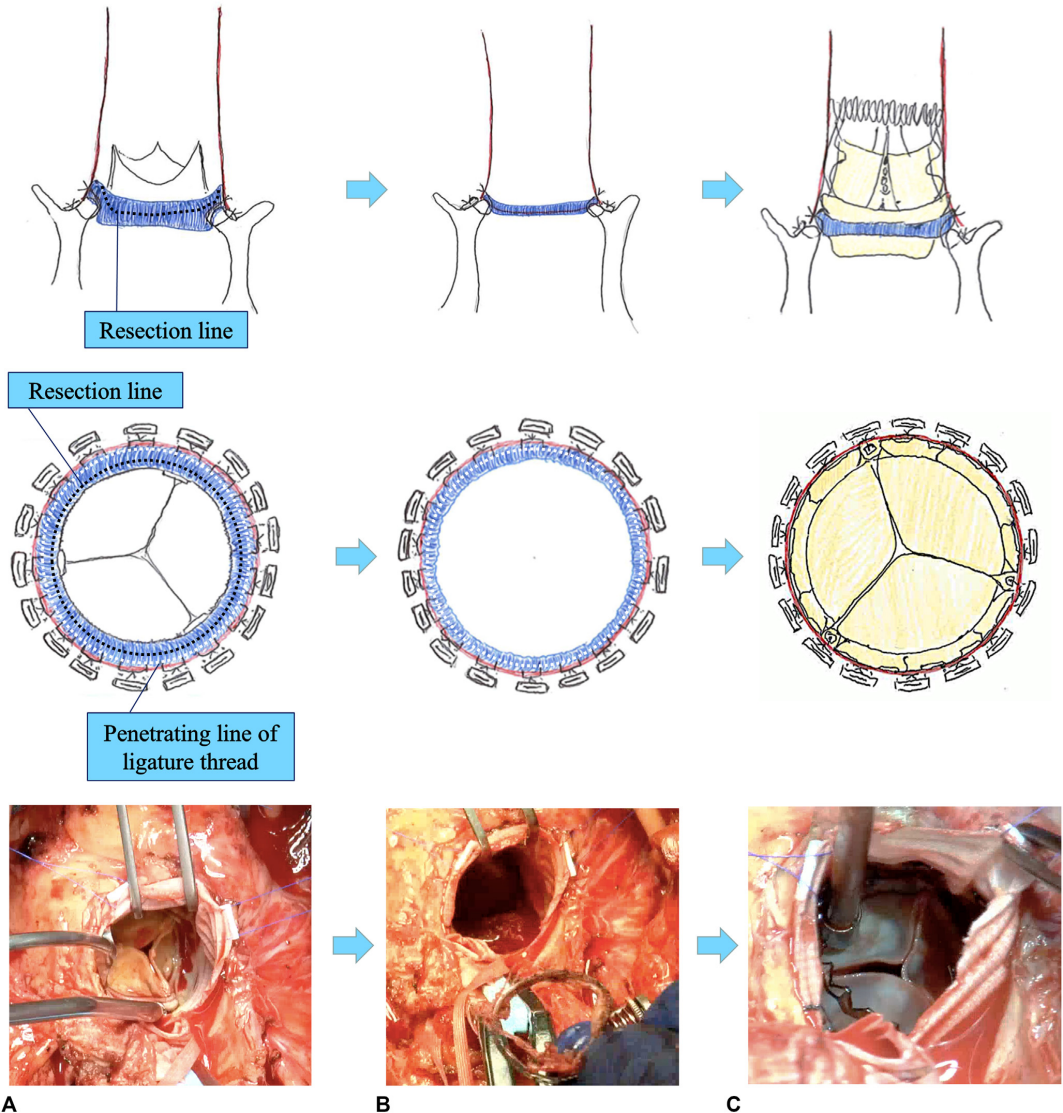
A, Degenerated Carpentier-Edwards Perimount prosthesis (*CEP*). B, Excision of Elgiloy and polyester band. C, Sutureless aortic valve implanted in the CEP cuff. (The *red* and *blue* structures indicate the prosthetic graft and prosthetic valve cuff, respectively. The *white dotted line* shows the penetrating line of ligature thread. The *black dotted line* shows the resection line of the prosthetic valve.)

A 76-year-old man underwent a modified Bentall procedure in October 2010 for treatment of aortic regurgitation and annuloaortic ectasia. During that initial surgery, a bioprosthetic stented conduit, composed of a stented Carpentier-Edwards Perimount (CEP) 21-mm bioprosthesis (Edwards Lifesciences) and a Gelweave polyester straight prosthetic graft 26 mm in size (Terumo Cardiovascular Systems Inc), was placed directly by suturing to the native aortic annulus using everting Nespolen 2–0 mattress pledgeted sutures (Figure 1, *A*).

Thirteen years later, the patient presented with decompensated heart failure (New York Heart Association functional class III). Transesophageal echocardiography indicated a heavily degenerated biological aortic valve prosthesis with severe trans and paravalvular leaks documented (mean pressure gradient, 23 mm Hg; ejection fraction, 74%; aortic valve area, 0.9 cm^2^). Building on the findings of the preceding surgery, the likelihood of a paravalvular leak was initially considered low. However, multiple echocardiologists provided an unequivocal diagnosis of a leak. The European System for Cardiac Operative Risk Evaluation II calculation was 10.5%. A redo aortic valve replacement (AVR) procedure through a median sternotomy was planned. Due to suspicion that the paravalvular leak was severe, a valve-in-valve strategy using a conventional stented prosthesis was ruled out and redo AVR surgery was scheduled for June 2023.

To initiate the redo surgery, cardiopulmonary bypass was established with use of the right axillary artery for blood supply and the right jugular and right femoral veins for blood drainage. Previously developed adhesions around the prosthetic graft were significant, thus the incision for the prosthetic graft was made at a position higher than typical for AVR. Examination of the aortic bioprosthesis showed it to be completely degenerated as well as calcification of the 3 leaflets of the CEP valve, although there were no morphological signs of endocarditis (Figure 1, *A*). Additionally, there were no fissures around the prosthetic valve that could have led to regurgitation, as also indicated in echocardiogram findings.

The 3 leaflets were removed entirely and pannus tissue attached to the prosthetic valve was completely detached. The metal portion becomes exposed in proximity to the stent post. The exposed Elgiloy and polyester bands (Figure 1, *B*) were carefully excised using a Harmonic scalpel and normal sharp scalpel to prevent damage to prior anastomotic threads and sutures, including the pledget. The sewing cuff and pledget of the CEP prosthesis were left in place. Cautious release of the self-expandable sutureless aortic valve (SAV) (Perceval aortic pericardial heart valve [size S]; Corcym) was performed inside the composite graft of the remnant cuff of the CEP valve (Figure 1, *C*). Additionally, it was confirmed that the prosthetic valve did not impede the coronary button.

The prosthesis was successfully implanted with a crossclamp time of 66 minutes and cardiopulmonary bypass time of 96 minutes. Immediate postoperative transesophageal echocardiography control excluded paravalvular leakage and showed a satisfactorily positioned prosthesis. No evidence of arrhythmia such as atrioventricular blockage was observed from the time of cardiopulmonary bypass weaning. At the time of discharge, dyspnea was improved to New York Heart Association functional class I. There were no issues identified in postoperative computed tomography scan results. Four weeks postoperatively, echocardiography indicated no paravalvular leak, and the peak/mean gradient measured 18/9.4 mm Hg. This favorable outcome remained consistent with no increase in the pressure gradient observed at the 5-month follow-up. The patient provided informed written consent for publication of the case data (IRB/ERB TGE02016-025; September 12, 2022).

## Discussion

Findings obtained in this case demonstrate the feasibility of implanting an SAV in patients who previously underwent root replacement surgery without need for replacing the root or performing coronary artery reimplantation. Reoperative aortic root surgery with a prosthetic valve and coronary artery reconstruction is extremely risky. Although previous studies have emphasized the clinical rationale for such procedures, mortality rates ranging from 18% have been noted.^1^ Another report noted that implantation of an SAV helped avoid redo aortic root replacement, which would have been a prolonged and challenging operation.^2^

Gallegos and colleagues^3^ conducted a study on the Bentall procedure employing the SAV as the primary surgical prosthesis. The implantation of the prosthetic valve followed the suturing of the prosthesis and reconstruction of the coronary arteries. Subsequent to these preparatory measures, the SAV was implanted using standard techniques.^3^ Additionally, Dhanekula and colleagues^4^ provided a summary of reoperations involving SAVs that included good outcomes in 4 cases after Bentall surgery, although details regarding those specific cases were not provided. To the best of our knowledge, the present is the first report of insertion of an SAV in a patient who had previously undergone Bentall surgery with a composite graft, with the cuff and sutured pledget of the previous prosthetic valve left in place. For this case, the composite graft was opened, then the existing CEP valve was carefully excised using precise techniques to preserve surrounding structures, previous sutures, and pledget integrity. Subsequently, an SAV was inserted within the existing composite graft.

## Conclusions

The present results demonstrate that redo AVR with an SAV can help to avoid a redo aortic root replacement procedure.

## Conflict of Interest Statement

The authors reported no conflicts of interest.

The *Journal* policy requires editors and reviewers to disclose conflicts of interest and to decline handling manuscripts for which they may have a conflict of interest. The editors and reviewers of this article have no conflicts of interest.
